# Association of TIM-3 with BCLC Stage, Serum PD-L1 Detection, and Response to Transarterial Chemoembolization in Patients with Hepatocellular Carcinoma

**DOI:** 10.3390/cancers12010212

**Published:** 2020-01-15

**Authors:** Maria Tampaki, Evangelos Ionas, Emilia Hadziyannis, Melanie Deutsch, Katerina Malagari, John Koskinas

**Affiliations:** 1 Department of Internal Medicine, Medical School of Athens, Hippokration Hospital, 115 27 Athens, Greece; dc_martam@hotmail.com (M.T.); emhadzi@med.uoa.gr (E.H.); meladeut@gmail.com (M.D.); 2 Department of Gastroenterology, G. Gennimatas General Hospital, 115 27 Athens, Greece; vag186@yahoo.gr; 3 Department of Radiology, Athens University, Attikon Hospital, Chaidari, 124 62 Athens, Greece; kmalag@otenet.gr

**Keywords:** HCC, TIM-3, TACE, immune checkpoint

## Abstract

Considering the increasing importance of immune checkpoints in tumor immunity we investigated the clinical relevance of serum T-cell immunoglobulin and mucin domain-3 (TIM-3) in patients with hepatocellular carcinoma (HCC). Serum TIM-3 levels were measured and their association with HCC stage and the detection of serum programmed death ligand-1 (PD-L1) were assessed. In patients submitted to transarterial chemoembolization (TACE), pre- and 1-week post-treatment TIM-3 levels were also evaluated. We studied 53 HCC patients with BCLC stages: 0 (5.7%), A (34%), B (32.1%), C (22.6%), and D (5.7%). The patients with advanced HCC (BCLC C) had significantly higher TIM-3 levels than patients with BCLC A (*p* = 0.009) and BCLC B (*p* = 0.019). TIM-3 levels were not associated with HCC etiology (*p* = 0.183). PD-L1 detection (9/53 patients) correlated with TIM-3 levels (univariate analysis, *p* = 0.047). In 33 patients who underwent TACE, post-treatment TIM-3 levels (231 pg/mL, 132–452) were significantly higher than pre-TACE levels (176 pg/mL, 110–379), (*p* = 0.036). Complete responders had higher post-TACE TIM-3 levels (534 pg/mL, 370–677) than partial responders (222 pg/mL, 131–368), (*p* = 0.028). Collectively, TIM-3 may have a role in anti-tumor immunity following TACE, setting a basis for combining immunotherapy and chemoembolization.

## 1. Introduction

Hepatocellular carcinoma (HCC) is the fifth most common cancer globally and develops mainly in the presence of cirrhosis, as a result of chronic viral hepatitis or other chronic liver disease [1]. Despite its high prevalence, the available systematic treatments are indicated only in the advanced stages and were quite limited until recently [1,2]. The deeper understanding of immune checkpoints’ role in the development of immune tolerance against HCC has led to the entrance of immune checkpoint inhibitors in the therapeutic landscape [3,4]. Specifically, it has been proven that the upregulated expression of immune inhibitory molecules such as programmed death-1 (PD-1) by antigen presenting cells (APCs) and by T cells in the tumor microenvironment has a critical role in the establishment of a defective immune response against HCC [3,4]. This effect is further enhanced through the secretion of activating ligands such as PD-1-ligand (PD-L1) by tumor cells and by tumor associated macrophages (TAMs). Nivolumab, an anti-PD-1 monoclonal antibody, is the first immunotherapeutic agent that was approved by FDA as a second-line treatment for advanced HCC [1,5]. Its anti-tumor efficacy is based on the blockade of PD-1/PD-L1 axis and the promotion of tumor-specific T cell activation [5]. Following nivolumab, pembrolizumab, another anti-PD-1 agent, was approved for the treatment of HCC, while numerous agents targeting different immune checkpoints such as cytotoxic T-lymphocyte antigen-4 (CTLA-4), PD-1, and PD-L1 are currently under evaluation as monotherapy or as combination treatment in various studies [6]. However, the complexity and interaction of these immune pathways should be taken into consideration especially in cases of treatment combination or treatment failure. In fact, the increased expression of inhibitory immune checkpoints such as T-cell immunoglobulin and mucin domain-3 (TIM-3) has been proven to affect the efficacy of anti-PD-1 agents [7,8]. Specifically, TIM-3, through the interaction with its ligand, galectin-9, downregulates the population and activity of T helper 1 (Th1) cells and promotes the “exhaustion” of T cells in many types of cancer [9,10]. The expression of TIM-3 on various innate and adapted effector cells such as dendritic cells, macrophages, natural killer cells, and natural killer T cells [9] indicates its involvement in a rather complex and vast range of immune pathways that interact with the PD-1/PD-L1 axis. According to a recent study, higher serum levels of TIM-3 correlated with advanced HCC stage and poor prognosis in patients with HBV-related HCC [11]. It is possible that the TIM-3/gal-9 axis plays a crucial role in the development of immune tolerance against HCC cells and its targeting could have an impact on the efficacy of existing immunotherapies.

On the other hand, the use of locoregional treatments and specifically transarterial chemoembolization (TACE), has been established in the management of patients with intermediate stages of HCC and its efficacy has been supported by large cohorts [12]. Although the potency of TACE in HCC treatment is unquestionable, its effect on the immune response towards HCC is not fully elucidated. The activation of CD4+ cells and anti-tumor CD8+ cells following TACE has been described in previous studies and more importantly it has been associated with better response to treatment [13]. It is possible that the acute inflammation and the liberation of antigens caused by the necrosis of the tumor cells greatly enhance the response of the previously tolerant immune system. However, there is evidence that the extent of this immune response may be undermined by the increased expression of immunosuppressive molecules such as PD-L1 or TIM-3 in the tumor microenvironment. [14] This effect is particularly interesting considering that the combination of immunotherapy with TACE is under investigation in several studies [4].

The aim of this study is to investigate the association between serum TIM-3 (sTIM-3) levels and the stage of HCC and identify any correlations between the levels of sTIM-3 and serum PD-L1 (sPD-L1). Moreover, we studied the change of sTIM-3 values in patients with HCC who were submitted to TACE and their correlation to therapeutic response. Our results suggest for the first time that sTIM-3 levels are strongly associated with Barcelona Clinic Liver Cancer (BCLC) stage in patients with various types of underlying liver disease. Furthermore, sTIM-3 levels are significantly increased following treatment with TACE, while patients with complete therapeutic response had higher post-treatment values as compared with those who had partial response.

## 2. Results

### 2.1. Patient Characteristics

Fifty-three patients with HCC of various etiologies were prospectively enrolled in this study. The median age was 76 ± 11 years, while most of the patients (71.7%) were males. All patients were cirrhotic and 10/53 (18.9%) had decompensated disease. The most prevalent cause of hepatic disease was viral hepatitis in 45/53 (84.9%) patients. Baseline BCLC stages, Child–Pugh scores, and additional tumor characteristics are described in Table 1.

### 2.2. Serum TIM-3 Values Are Associated with HCC Stage

**Figure 1:**
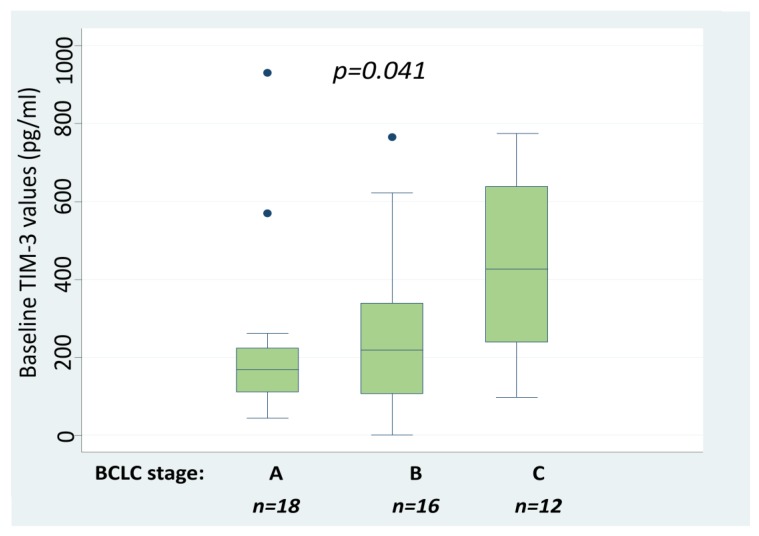
Baseline sTIM-3 values according to BCLC stage in 46 HCC patients. Kruskall–Wallis test was applied to compare median values per BCLC stage A, B and C. BCLC; Barcelona Clinic Liver Cancer, sTIM-3; serum T-cell immunoglobulin and mucin domain-3, HCC; Hepatocellular carcinoma.

Baseline sTIM-3 levels were detectable in 52/53 (98%) HCC patients. The median values of sTIM-3 in patients according to BCLC stage were 132 pg/mL (Q25–75: 93–143 pg/mL) in BCLC 0, 171 pg/mL (Q25–75: 113–232 pg/mL) in BCLC A, 218 pg/mL (Q25–75: 108–339 pg/mL) in BCLC B, 425 pg/mL (Q25–75: 266–633 pg/mL) in BCLC C, and 2671 pg/mL (Q25–75: 234–5000 pg/mL) in BCLC D. Among the three patients with BCLC D, all had advanced liver disease (two with Child–Pugh score B and 1 with C) and performance status > 2 with diffuse multinodular HCC: two with main portal vein invasion and one with metastatic bone disease. Kruskal–Wallis test was applied to compare values between stages A–C. Patients with BCLC 0 and BCLC D were excluded from the analysis due to the small number of patients in each group. Outliers were also excluded. Serum TIM-3 levels differed significantly between BCLC stages A, B, and C with higher levels detected in more advanced stages (*p* = 0.041, Figure 1). Additionally, a Dunn test was performed as a post-hoc analysis to determine which pairs of BCLC groups had statistically different sTIM-3 values from each other (Table 2). The patients with advanced HCC (BCLC C) had significantly higher sTIM-3 values than patients with BCLC A (*p* = 0.009) and BCLC B (*p* = 0.019). Interestingly, all three patients with sTIM-3 levels close to the upper highest limit of detection had BCLC stage ≥ C, while the patient with undetectable sTIM-3 levels had BCLC stage B. The above findings suggest that higher sTIM-3 values are strongly associated with advanced HCC staging. On the other hand, sTIM-3 median values did not differ significantly between patients with viral (186 pg/mL Q25–75: 115–312) and patients with non-viral HCC etiology (262 pg/mL Q25–75: 132–929) (Mann–Whitney test, *p* = 0.183). Moreover, the levels of sTIM-3 did not correlate with Child–Pugh score (Kruskal–Wallis test, *p* = 0.354).

### 2.3. Association of sTIM-3 Levels with the Detection of sPD-L1

**Figure 2:**
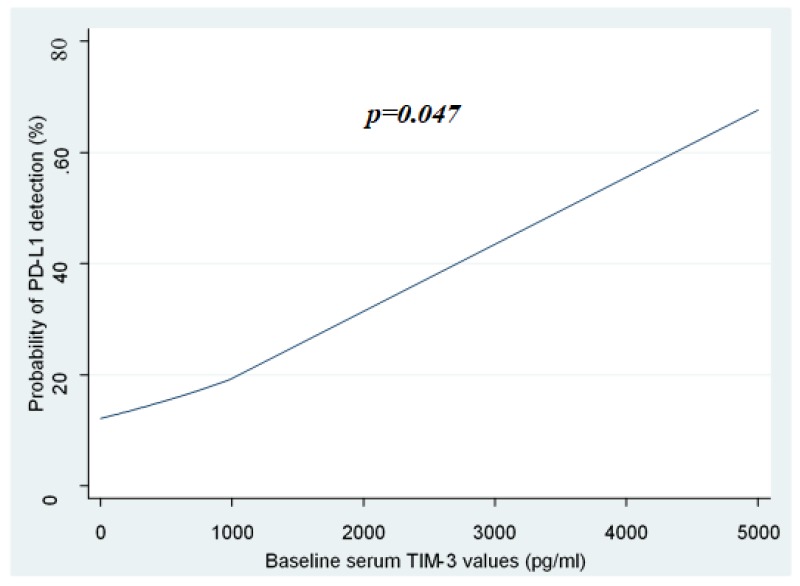
Association of sTIM-3 values with the probability of s PD-L1 detection. Higher sTIM-3 values were associated with a higher probability of sPD-L1 detection. Univariate logistic regression was used as a statistical model to show the probability of detection/no-detection of PD-L1 versus sTIM-3 values. sTIM-3; serum T-cell immunoglobulin and mucin domain-3, sPD-L1; serum Programmed death ligand-1.

Since both PD-1/PD-L1 immune checkpoint and TIM-3/gal-9 axis are believed to play a significant role in T-cell exhaustion and immune tolerance against cancer [7,8], we hypothesized a possible interaction between the two pathways. Serum PD-L1 was detected only in 9/53 (17%) patients. Among these patients, 4/9 (44%) had Child–Pugh score A and 5/9 (56%) had Child–Pugh score B, while 8/9 (89%) had chronic viral hepatitis-related HCC. Interestingly, 8/9 (89%) patients with detectable sPD-L1 levels (median: 3.51 ng/mL, range: 1.34–18.48 ng/mL) had advanced HCC (BCLC C) and 1/9 (11%) had BCLC A (Table 3). Regarding the detection of PD-L1 only in 17% of the patients, we investigated whether its detection or non-detection is affected by sTIM3 levels. Multivariate logistic regression did not reveal any significant association between the probability of serum PD-L1 detection and sTIM-3 values (Table 3). However, the univariate logistic regression model showed that baseline sTIM3 levels were significantly associated with the probability of sPD-L1 detection (*p* = 0.047, Figure 2). In other words, univariate logistic regression showed that higher sTIM-3 values were associated with a higher probability of sPD-L1 detection. Although not proven by multivariate analysis, this association may be the result of simultaneous activation of both immune checkpoints in cases of HCC, resulting in a strong immunosuppressive effect, especially in cases of advanced cancer. Serum PD-L1 levels did not correlate significantly to HCC etiology (Mann–Whitney *U* test, *p* = 0.732), Child–Pugh score (Kruskal–Wallis test, *p* = 0.488) and HCC stage (Kruskal–Wallis test, *p* = 0.063) probably due to the small number of patients with detectable sPD-L1.

### 2.4. Serum TIM-3 Levels Are Increased Significantly 1-Week Post-TACE

Thirty-three patients of the study population with different stages of HCC (BCLC A 12 (36.4%), BCLC B 15 (45.5%), BCLC C 6 (18.1%)) were submitted to TACE. Serum TIM-3 levels were measured in this specific group at baseline and at the first week post-treatment.

**Figure 3:**
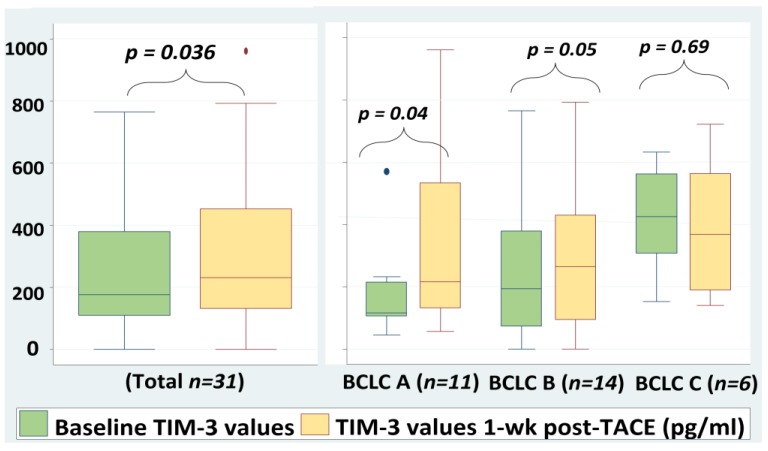
Serum TIM-3 values pre- and 1-week post-TACE. Wilcoxon test was used for the comparison of sTIM-3 values. Outliers were excluded from the analysis. sTIM-3; serum T-cell immunoglobulin and mucin domain-3, TACE; transarterial chemoembolization, BCLC; Barcelona Clinic Liver Cancer.

At baseline, 32/33 (97%) patients had detectable sTIM-3 levels with median values of 176 pg/mL (Q25–Q75: 111–379 pg/mL). Wilcoxon test was used for the comparison of pre- and post-TACE sTIM-3 values. Interestingly, following TACE treatment, sTIM-3 levels increased significantly with median values of 231 pg/mL, (Q25–75:132–452 pg/mL, *p* = 0.036, Figure 3). Based on these results, we assume that TACE induced an immediate immune response following the necrosis of tumor cells that caused a reactive increase of serum TIM-3 levels at the first week post-treatment.

Four patients of this group had detectable sPD-L1 levels (median: 7.45 ng/mL, range: 2.15–13.7 ng/mL) before TACE, but there was no significant change at the first week post-treatment (median: 10.8 ng/mL 2.57–19.6 ng/mL) (Wilcoxon test). This could be explained by the small patient sample and the low detection rate of sPD-L1 in this population.

### 2.5. Correlation between Post-TACE sTIM-3 Levels and Response to Treatment

The response of the 33 patients who were submitted to TACE was evaluated according to Modified Response Evaluation Criteria in Solid Tumors (mRECIST) criteria, at the first month post-treatment either with a Magnetic Resonance Imaging (MRI) or a Computerized Tomography (CT) scan. Among these patients, 27/33 (81.8%) had a partial response to TACE, 5/33 (15.2%) had a complete response, and 1/33 (3%) had progressive disease at the time of evaluation. Among the patients with complete response, one patient had HCC BCLC stage A, two BCLC B, and two BCLC C. The response to TACE did not correlate to baseline BCLC stage (*p* = 0.3). Moreover, three out of four patients with detectable PD-L1 levels (75%) had a partial response to TACE and one responded completely (25%). There was no correlation between the levels of sPD-L1 and the response to treatment (Mann–Whitney *U* test, *p* = 0.263).

**Figure 4:**
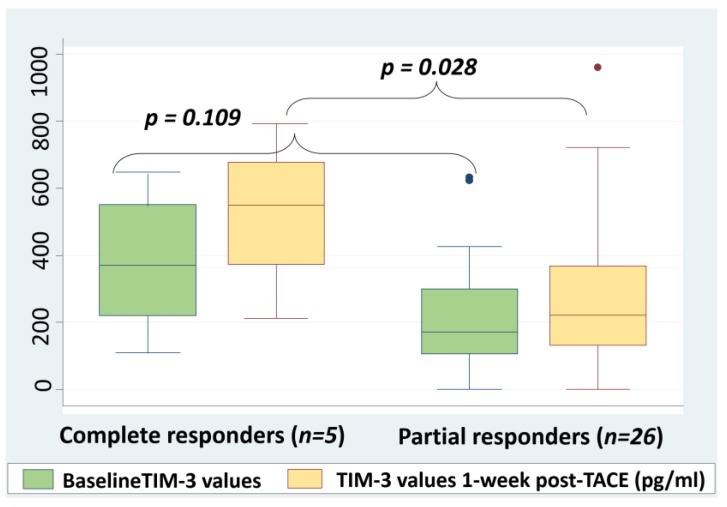
Pre- and post-TACE sTIM-3 values according to treatment response based on Modified Response Evaluation Criteria in Solid Tumors (mRECIST) criteria. Mann–Whitney *U* test was applied to compare sTIM-3 values. Outliers were excluded from the analysis. sTIM-3; serum T-cell immunoglobulin and mucin domain-3, TACE; transarterial chemoembolization.

Baseline and 1-week post-TACE sTIM-3 levels were compared between the patients with complete response and those with partial response. Mann–Whitney *U* test was applied for this comparison. There was no statistically significant difference between the baseline sTIM-3 levels of complete responders (368 pg/mL, Q25–75:223–534 pg/mL) and partial responders (173 pg/mL, Q25–75:106–299 pg/mL) (*p* = 0.109). However, the median post-TACE levels were significantly higher in the group of complete responders (534 pg/mL, Q25–75:370–677 pg/mL) when compared to the median levels of the group of partial responders (222 pg/mL, Q25–75:131–368 pg/mL, *p* = 0.028, Figure 4). Despite the limitation of the small number of complete responders, a comparison between the median change ratios of sTIM-3 levels was performed in the two groups. The median change ratio for the sTIM-3 values of complete responders (0.481) was higher than the respective median ratio of partial responders (0.193), however the difference was not statistically significant (Mann–Whitney *U* test, *p* = 0.527). Although the number of patients is limited, the above results could indicate that complete response to treatment may be associated with higher post-treatment sTIM-3 levels when compared to partial response due to the more extensive tumor necrosis, the induction of a stronger inflammatory response, and finally the higher expression of inhibitory molecules such as TIM-3.

## 3. Discussion

This is the first time that soluble TIM-3 values are positively associated with the stage of HCC in patients with various types of underlying liver disease. Furthermore, the effect of TACE on sTIM-3 values has not been previously studied. According to our findings, treatment with TACE causes a significant increase of sTIM-3 levels at the first week post-treatment, while this increase is more prominent in patients with complete response.

It is well known that the development of HCC is usually preceded by chronic inflammation and cirrhosis. In this continuous inflammatory state, the physiological functions of T cells are gradually affected, while the antigen presenting cells (APCs) express immune checkpoint inhibitors such as PD-1 and TIM-3 that further suppress the immune response against cancer cells [15]. In parallel, there is an increase of T-regulatory cells (Tregs) that further promote the development of immune tolerance against HCC through the downregulation of T effector cells [15]. Interestingly, even the efficacy of chemotherapy with sorafenib may be negatively affected by the immunological microenvironment of the tumor, as shown in patients with higher systemic immune-inflammation index (SII) score, and higher neutrophil-to lymphocyte ratio (NLR) [16]. In order to reverse the established immune exhaustion and tolerance, the main immunotherapeutic approaches against HCC include (i) adoptive immunotherapy that includes HCC-epitopes immunized cells which recognize and act against cancer cells; (ii) indirect immunological strategies such as immune checkpoint blockade with monoclonal antibodies, cytokines, and cancer vaccines; (iii) indirect non immunological strategies such as antigen-encoding mRNA, metronomic chemotherapy, and oncolytic viruses [15].

The TIM-3/gal-9 pathway is considered to have a critical role in immune suppression and immune escape for many types of cancer including HCC and it is gaining ground as an emerging target for future immunotherapy [17,18,19,20]. Additionally, according to a recent study, higher TIM-3 expression on circulating T cells of HCC patients was associated with lower response rates to immune checkpoint-inhibitor treatment (PD-1/PD-L1 blockade) [21]. Moreover, the downregulation of PD-1/PD-L1 axis by immunotherapy appears to cause an upregulation of TIM-3 expression on T cells, resulting in inadequate treatment responses and immunological escape of the tumor [22]. In our study, higher sTIM-3 levels were found to correlate with advanced stages of HCC and thus, poorer prognosis. These findings confirm previous observations that sTIM-3 levels are independently associated with the overall survival of patients with HBV-related HCC [11]. A possible interpretation of the above results is that more advanced stages of HCC are associated with a more intense palsy effect on the anti-tumor immune response. Specifically, the increased tumor burden causes an overproduction of tumor antigens that further promotes T-cell exhaustion through TIM-3 overexpression on specific CD8+ T cells. Interestingly, median sTIM-3 levels were very high in patients with BCLC D. The three patients in this group had advanced HCC with extensive tumor burden that could possibly explain the detection of high sTIM-3 values, although the patient number is limited. Based on the above, sTIM-3 may be a useful circulating biomarker for HCC staging and prognosis. Additional studies are needed to investigate whether baseline sTIM-3 levels could predict HCC susceptibility to immune checkpoint-inhibitors such as nivolumab or pembrolizumab in order to individualize treatment choice in advanced stages.

Following the emergence of immunotherapy in the treatment of HCC, several studies have shown a correlation between serum PD-1/PD-L1 levels, HCC aggressiveness, and patient prognosis [23,24]. Nevertheless, in our study population, sPD-L1 levels were below the lowest detection limit in most of the patients. When detected, the median levels (3.51 ng/mL, 1.34–18.48 ng/mL) were higher than the respective values (<0.8 ng/mL) of healthy controls as measured in a recent study [23]. The low detection rate of sPD-L1 in our study (17%) could be explained by the fact that PD-L1 is rarely expressed in neoplastic HCC cells (17% expression in histological samples) [25]. On the contrary, TIM-3 was detectable in the serum of 52/53 (98%) HCC patients. This result could indicate a more crucial role of the TIM-3/gal-9 pathway in the development of immune tolerance against HCC when compared to the PD-1/PD-L1 axis. Consequently, the inclusion of TIM-3/gal-9 blockade in the treatment of HCC could be very promising. In fact, the efficacy and safety of anti-TIM-3 monoclonal antibodies as immune treatment for different types of cancer is currently evaluated in preclinical studies [9,16].

Considering the interaction between the two immune pathways, a significant association between the levels of sTIM-3 and the probability to detect sPD-L1 was observed in the univariate logistic regression in our study, while multivariate analysis failed to prove any correlation. In a previous publication, simultaneous expression of TIM-3 and PD-1 on specific CD8+ T cells led to more severe CD8+ T cell exhaustion and expression of immunosuppressive cytokines in cases of chronic viral expression [26]. Similarly, the co-expression of TIM-3 and PD-1 on anti-tumor specific cells had a significant impairing effect on the function of T cells in cases of melanoma [27]. Our knowledge on the interaction between the two immune checkpoint inhibitors in HCC is very limited and remains to be investigated. However, since the activation of TIM-3 pathway may be a barrier to effective PD-1/PD-L1 blockade [21], it could be assumed that double blockade of both immune pathways will maximize the therapeutic effect and prevent the development of adaptive immune resistance by the tumor [28].

**Figure 5:**
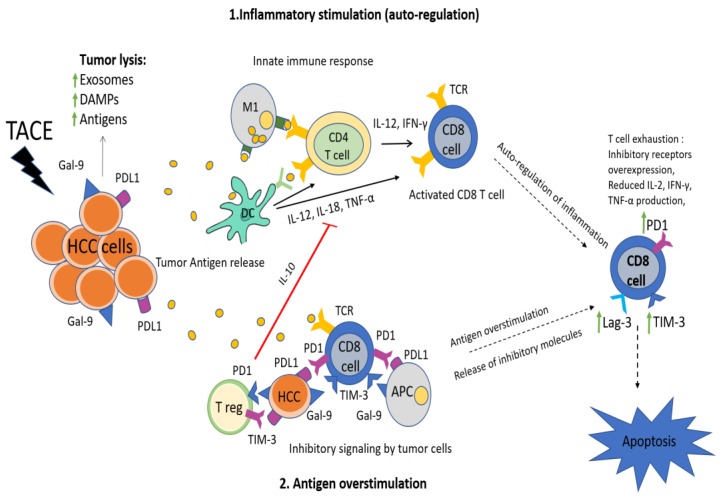
The “2-hit” hypothesis: (**1**) excessive inflammation induced by tumor lysis following TACE is autoregulated and self-restricted by expression of co-inhibitory receptors (TIM-3, PD-1, Lag-3) on T cells, (**2**) T-cell overstimulation by tumor-related antigens and inhibitory signaling by released tumor cells leads to T-cell exhaustion defined by poor effector function and expression of co-inhibitory receptors such as PD-1 and TIM-3, upregulation of T reg cells and reduced inflammatory cytokine production. Both pathophysiological mechanisms result in increased TIM-3 expression on the surface of CD8+ T cells and eventual depletion of the anti-tumor immune response. HCC; hepatocellular carcinoma, TACE; transarterial chemoembolization, NK; natural killer, APCs; antigen presenting cells, TIM-3; T cell immunoglobulin and mucin domain-3, Lag-3;Lymphocyte-activation gene-3, PD-1; programmed death 1, PD-L1; programmed death 1 ligand, IL; interleukin, IFN-γ; interferon-γ, TNF-α; tumor necrosis factor-alpha, T reg; t regulatory, DC; dendritic cells, M1; macrophage, DAMPs; damage-associated molecular patterns.

As previously mentioned, the pathophysiology of the immune response following the reduction of tumor burden by TACE is not yet fully understood. It has been shown that the necrosis and ischemia caused by TACE initially induce the production of acute phase cytokines such as interleukin (IL)-6 and IL-22, while in later phases immunosuppressive cytokines such as IL-10 are increased [29]. Moreover, a decrease of T regulatory (T reg) cells has been observed following TACE, indicating the restoration of immune function after treatment [30]. In our study, TACE induced a significant raise of sTIM-3 levels at the first week post-treatment. A possible explanation for this finding is the reactive increase of TIM-3 expression by T cells as a negative feedback to intense immune stimulation following tumor necrosis (Figure 5). Under normal conditions, TIM-3 is expressed on the surface of T cells in order to downregulate the inflammatory response and restore the homeostasis of the immune reaction. In fact, the physiological role of TIM-3 is affected and downregulated in cases of autoimmune disease [31]. In this context, TIM-3 expression may also increase reactively in order to downregulate the previous immune activation against tumor antigens [32,33]. Additionally, the post-TACE TIM-3 increase could be the result of immediate HCC antigen release following tumor necrosis that causes excessive and continuous T cell stimulation, leading to gradual overexpression of inhibitory immune receptors such as TIM-3 and PD-1 and finally resulting in further immune exhaustion. Similar observations have been reported in other types of cancer, showing an increase of PD-L1 and TIM-3 expression following chemotherapy-induced tumor necrosis [34,35]. Based on the above, the increase of TIM-3 levels following TACE could be summarized in the “2-hit” hypothesis: (a) the upregulation of TIM-3 expression in order to suppress the inflammation caused by tumor necrosis and (b) the exhaustion of T cells following tumor-antigen overstimulation (Figure 5). Given the fact that several trials combining TACE and immunotherapy are currently ongoing [4], the use of markers such as TIM-3 to define the tumor immune profile following TACE, could be very useful in order to predict tumor susceptibility to sequential immunotherapy. It is possible that the administration of immunotherapy after TACE could prolong and maximize the immune activation against HCC by preventing T cell exhaustion, although additional studies are required to elucidate this hypothesis.

Finally, an interesting finding in our study is the detection of higher post-treatment sTIM-3 levels in five patients with complete response to TACE (mRECIST criteria), comparing to 26 patients with partial response. Regarding the change ratios of sTIM-3 levels, complete responders had a higher median change ratio than partial responders (48.1% versus 19.3%), but this difference was not statistically significant. Further investigation is required to confirm this association since it is based on a small number of patients. Despite the above limitations, higher post-TACE sTIM-3 levels in complete responders may be attributed to the greater extent of tumor infiltration by TIM-3+ T lymphocytes in patients with better response to treatment. This finding has been previously described in cases of complete response to chemotherapy in other types of cancer [36]. Furthermore, a recent study in HCC patients has shown that yttrium-90 (Y90)-radioembolization (RE) induces an increase of PD-1+/TIM-3+ CD8+ T cells at 3 months post-treatment. This increase was found to be more prominent in sustained responders when compared to transient or non-responders [37]. The sustained responders to Y90-RE had increased expression of inflammatory activating markers and TNF-α on tumor infiltrating T cells and peripheral blood mononuclear cells (PBMCs) after treatment. The heightened expression of TIM-3 on the T cells of these patients was associated with the previous mounted immune activation following successful tumor necrosis. In the same context, in cases of successful treatment with TACE, more extended tumor lysis in complete responders in parallel with the excessive release of tumor antigens promotes the higher expression of TIM-3 when compared to partial responders. Consequently, the higher post-treatment sTIM-3 levels in complete responders in our study may be related to the fact that complete tumor necrosis could induce a more intense inflammatory response resulting in a greater TIM-3+ T cell tumor infiltration and a higher TIM-3 expression when compared to partial tumor necrosis. If this observation is confirmed in larger studies, the administration of adjuvant immunotherapy in such cases could optimize the therapeutic outcome.

## 4. Materials and Methods

### 4.1. Selection of Patients

Patients with a confirmed diagnosis of HCC (radiological or histological), presenting at the Academic Department of Internal Medicine of Hippokrateio General Hospital in Athens between January 2018 and December 2018 were included in this prospective study. All patients gave their written informed consent prior to study inclusion. The study was approved by the Scientific and Ethics Board of Hippokrateio General Hospital (approval code; 42/6-6-13). The exclusion criteria included: patients younger than 18 years old, a history of other types of cancer or a history of liver transplantation.

HCC staging was performed according to the BCLC criteria [1]. Child–Pugh Score (CPS) [38] was calculated based on clinical examination and patients’ laboratory parameters and imaging techniques findings. Transarterial chemoembolization was performed by the same expert radiologist with doxorubicin-eluting-bead embolization in all the patients. The response to treatment was evaluated by using the mRECIST criteria [39], based on Magnetic Resonance Imaging or Computed Tomography scan.

### 4.2. Blood Sampling and Measurements

Blood samples were collected from each patient at baseline visit. Additional blood samples were obtained by the patients who were submitted to TACE, at the first week post-treatment. The serum tubes were centrifuged at 3000 rpm for 10 min at 4 °C and then aliquoted and stored at −80 °C. The laboratory measurements were performed at the Laboratory of the Academic Department of Internal Medicine of Hippokrateio General Hospital in Athens.

Serum TIM-3 was measured using a commercially available Enzyme-linked immunosorbent Assay for quantitative detection of human TIM-3. (ELISA; TIM-3 Human ELISA Kit BMS 2219, Thermo Fisher Scientific Waltham, MA, USA) according to the recommendation of the manufacturer. The minimum detectable level of sTIM-3 was 35.3 pg/mL and the detection range was 78.1–5000 pg/mL. Serum PD-L1 was also measured with a commercially available ELISA kit (Human Protein Spindly CCDC99/SPD-L1 ELISA Kit, Abbexa Ltd., Innovation Centre, Cambridge Science Park, Cambridge, CB4 0EY, UK) according to the recommendation of the manufacturer. The minimum detectable level of s PD-L1 was 0.188 ng/mL and the test range was 0.313–20 ng/mL.

### 4.3. Statistical Analysis

The correlation between sTIM-3, sPD-L1 levels and BCLC stage of HCC was determined using the Kruskall–Wallis test, while BCLC stages 0 and D were excluded from the analysis due to the small number of patients. Dunn test was performed as a post-hoc analysis to determine differences of sTIM-3 levels between pairs of BCLC groups. Multivariate and univariate logistic regression was used as a statistical model to show the probability of detection/no detection of PD-L1 versus sTIM-3 values. The difference between pre- and post-treatment sTIM-3 and sPD-L1 levels in patients who were submitted to TACE was calculated with Wilcoxon test. The association of sTIM-3 and sPD-L1 levels with Child–Pugh score and the type of underlying liver disease was tested with Kruskall–Wallis test, and Mann–Whitney *U* test, respectively. The correlation between pre/post-TACE sTIM-3 levels and response to therapy according to mRECIST criteria was also assessed by using the Mann–Whitney *U* test. The median change ratios of sTIM-3 levels (post-treatment levels − baseline levels/baseline levels) were compared in complete and partial responders with Mann–Whitney *U* test. Outliers were excluded from the analysis. *p* values < 0.05 were considered to be significant. Continuous variables are shown as median and Q25–Q75 quartiles and categorical variables are reported as frequencies and percentages. Statistical analyses were performed with SPSS (version 22.0, IBM, New York, NY, USA).

## 5. Conclusions

According to the findings of this study, serum TIM-3 levels are associated with HCC stage in patients with various types of underlying liver disease. In addition, treatment with TACE causes an escalation of circulating TIM-3 levels, while higher post-treatment serum TIM-3 values were observed in patients with complete response comparing to patients with partial response. This is the first time that the clinical relevance of sTIM-3 levels is evaluated in patients with HCC who are submitted to TACE.

In view of the increasing research on combinations of locoregional treatments and immunotherapy, the effect of TACE on immune checkpoint pathways is of great interest. Additional studies are needed to shed light on the exact impact of sTIM-3 levels on the efficacy of immunotherapy following TACE in order to achieve better treatment responses. Furthermore, the detection of TIM-3 in the serum of all HCC patients could indicate that TIM-3/gal-9 pathway plays a more critical role than PD-1/PD-L1 axis in HCC immunity and may be a potential target for future HCC treatments. Since both pathways are involved in cancer-related immune exhaustion, double blockade of the PD-1/PD-L1 axis and TIM-3 pathway following locoregional treatments could promote the therapeutic outcome and improve the overall survival in patients with HCC.

## Figures and Tables

**Table 1:** Baseline patient characteristics.

Variables	
Age (median, range)	76, (30–88) years
Sex n, (%)	
Male	38, (71.7%)
Female	15, (28.3%)
BCLC stage n, (%)	
0	3, (5.7%)
A	18, (34%)
B	17, (32.1%)
C	12, (22.6%)
D	3, (5.7%)
Liver disease n, (%)	
HCV infection	28, (52.8%)
HBV infection	17, (32.1%)
NASH	4, (7.5%)
Alcoholic liver disease	2, (3.8%)
Other	2, (3.8%)
Child–Pugh score n, (%)	
A	33, (62.3%)
B	19, (35.8%)
C	1, (1.9%)
Tumor size n, (%)	
>5 cm	40, (75.4%)
≤5 cm	13, (24.5%)
Number of lesions n, (%)	
Single	16, (30.2%)
Multiple	37, (69.8%)
Patients submitted to TACE	33, (62.3%)
BCLC A	12, (36.4%)
BCLC B	15, (45.5%)
BCLC C	6, (18.1%)

HCV: hepatitis C virus; HBV: hepatitis B virus; NASH: non-alcoholic steatohepatitis; TACE: transarterial chemoembolization; BCLC: Barcelona Clinic Liver Cancer.

**Table 2:** Comparison of sTIM-3 values between pairs of BCLC groups. Dunn test was performed for post-hoc analysis. Patients with BCLC 0 and D were excluded due to small number of patients and outliers were also excluded from the analysis. Statistically significant differences are in bold. BCLC; Barcelona Clinic Liver Cancer, sTIM-3; serum T-cell immunoglobulin and mucin domain-3.

BCLC Stages (Median sTIM-3 Values, Q25–Q75 Values)	BCLC A (171 pg/mL, 113–232 pg/mL)	BCLC B (218 pg/mL, 108–339 pg/mL)
BCLC B (218 pg/mL, 108–339 pg/mL)	*p* = 0.364	
BCLC C (425 pg/mL, 266–633 pg/mL)	*p* = 0.009	*p* = 0.019

**Table 3:** Serum TIM-3 values and clinical characteristics in patients with detectable serum PD-L1. Univariate logistic regression was applied for the association of sTIM-3 values with the probability of sPD-L1 detection. sTIM-3; serum T-cell immunoglobulin and mucin domain-3, sPD-L1; serum programmed death ligand-1, BCLC; Barcelona Clinic Liver Cancer, TACE; transarterial chemoembolization, CR; complete response, PR; partial response, N/A; not applicable for the patients who were not submitted to TACE.

Patients with Detectable sPD-L1	Child–Pugh Score	BCLC Stage	Response to TACE	sTIM-3 Levels (pg/mL)
**Patient 1**	A	C	N/A	266
**Patient 2**	B	C	N/A	317
**Patient 3**	A	A	N/A	166
**Patient 4**	A	C	N/A	186
**Patient 5**	B	C	N/A	4.893
**Patient 6**	B	C	CR	562
**Patient 7**	B	C	PR	633
**Patient 8**	A	C	PR	153
**Patient 9**	B	C	PR	1.896
**Association of sTIM-3 values with the probability of sPD-L1 detection (univariate logistic regression):**				*p* = 0.047.
